# Extract from *Rosa* spp. as a Factor Influencing the Growth Rate of Coagulase-Negative *Staphylococcus* Strains

**DOI:** 10.3390/molecules30071443

**Published:** 2025-03-24

**Authors:** Lidia Piekarska-Radzik, Joanna Milala, Robert Klewicki, Michał Sójka, Dorota Żyżelewicz, Bożena Matysiak, Elżbieta Klewicka

**Affiliations:** 1 Institute of Fermentation Technology and Microbiology, Faculty of Biotechnology and Food Sciences, Lodz University of Technology, 171/173 Wólczańska St., 90-530 Lodz, Poland; 2 Institute of Food Technology and Analysis, Faculty of Biotechnology and Food Sciences, Lodz University of Technology, 4/10 Stefanowskiego St., 90-924 Lodz, Poland; joanna.milala@p.lodz.pl (J.M.); robert.klewicki@p.lodz.pl (R.K.); michal.sojka@p.lodz.pl (M.S.); dorota.zyzelewicz@p.lodz.pl (D.Ż.); 3 The National Institute of Horticultural Research, Department of Applied Biology, 96-100 Skierniewice, Poland; bozena.matysiak@inhort.pl

**Keywords:** *Staphylococcus* spp., cocci, antagonistic activity, growth dynamics, *Rosa* spp.

## Abstract

Coagulase-negative bacteria of the *Staphylococcus* genus are currently frequent food contaminants. The increase in antibiotic resistance means that these microorganisms are becoming the cause of many serious infections and toxications. Their resistance to routinely used chemical compounds has led to the search for alternative methods to combat food-borne pathogens. For this purpose, plant extracts rich in phenolic compounds are increasingly used. The aim of this study was to assess the effect of extracts obtained from the pseudo-fruits and flesh of *Rosa canina*, *Rosa rugosa* and *Rosa pomifera* ‘Karpatia’ on the growth dynamics of bacterial strains of the *Staphylococcus* genus (72-h co-culture; plate inoculation method). The conducted studies allowed us to conclude that extracts from *Rosa* spp. show high antistaphylococcal activity. However, it is not proportional to the dose used. *Rosa* spp. extracts already at concentrations of ¼ MIC limit the growth of the biomass of bacteria of the *Staphylococcus* genus. The above-described dependencies are very individual—strain-specific, not species-specific. However, based on SEM analysis, it can be observed that the antistaphylococcal mechanism of action of *Rosa* spp. extracts is associated with the coating of cell walls by the extracts and the disintegration of cell membranes, as a result of which the cells are destroyed.

## 1. Introduction

Food safety is the basic and most important criterion in the food industry. At the same time, it is one of the greatest challenges facing food producers [1]. Coagulase-negative staphylococci are a group of microorganisms that dominate fermented foods around the world. They are primarily used as starter cultures for the fermentation of meat products. They can also be starter cultures in the dairy industry. In recent years, there have also been reports confirming the use of *Staphylococcus* bacteria for soy fermentation.

Coagulase-negative staphylococci isolated from food are generally considered non-pathogenic microorganisms. However, the increase in antibiotic resistance in this group of microorganisms has led to them being increasingly isolated from skin infections of humans and animals. In addition, it should be remembered that they belong to the same genus as the species *Staphylococcus aureus*; therefore, they show some common mechanisms related to pathogenesis and the induction of infection [2,3]. It is also important that coagulase-negative staphylococci may be responsible for the synthesis of enterotoxins and potentially cause food poisoning [4].

Additionally, it is disturbing that the enterotoxins produced by them are characterized by resistance to high temperatures, pH, the action of proteolytic enzymes, freezing, and drying. They remain insensitive to enzymatic digestion in the human gastrointestinal tract. Although coagulase-negative staphylococci are among the strains very often present in food—much more often than coagulase-positive staphylococci—in the European Union countries, there is no obligation to determine their number [5].

Considering the progress of the spread of antibiotic resistance of strains of the *Staphylococcus* genus, alternative ways of limiting the growth of these microorganisms should be explored. One way to fight food pathogens may be plant extracts, which are widely used in the food industry [6]. Plant extracts rich in phenolic compounds show antagonistic potential in relation to pathogenic and undesirable microorganisms in food. Consequently, plant extracts can also be an alternative to traditionally used preservatives (antioxidant and antibacterial effects) [7].

The antimicrobial activity of phenolic compounds and their metabolic products is linked to their chemical structure, particularly the substituents in the phenolic ring [8]. Most literature data clearly indicate a significantly higher sensitivity to phenolic compounds in Gram-positive bacteria compared to Gram-negative species [9].

The primary mechanism of the antimicrobial action of phenolic compounds involves the deformation of the cell membrane structure, leading to potential ruptures due to structural instability. These ruptures increase membrane permeability, resulting in the loss of essential ions, nucleic acids, and amino acids necessary for bacterial growth and function [10,11]. Polyphenolic compounds in plant extracts also influence the expression of multiple genes related to virulence (e.g., the synthesis of *p*-fimbriae proteins, biofilm formation ability). In *Staphylococcus* species, changes in gene expression related to extracellular and intracellular protein synthesis (e.g., beetroot extract) have been observed, leading to increased membrane permeability [12,13,14]. Another mechanism involves the depolarization of bacterial membranes caused by plant extracts, which disrupts the ATP concentration within bacterial cells, preventing their further growth [15]. Phenolic compounds in plant extracts also act as pro-oxidative agents against microorganisms. Hydrogen peroxide, generated during the oxidation of polyphenolic compounds, exhibits bactericidal effects by inducing DNA fragmentation [16].

Many plant extracts exhibit anti-*Staphylococcus* properties, including those derived from: guava (*Psidium guajava* L.), sage (*Salvia officinalis* L.), rhamnus (*Ziziphus spina-christi*), mulberry (*Morus alba* L.) and olive (*Olea europaea* L.) at concentrations of 500–1250 µg/mL; roselle (*Hibiscus sabdariffa*), rosemary (*Rosmarinus officinalis*), clove (*Syzygium aromaticum*) and thyme (*Thymus vulgaris*) at concentrations of 1–20% (*w/v*); cumin (*Cuminum cyminum*), pomegranate (*Punica granatum*), clove (*Syzygium aromaticum*), thyme (*Thymus vulgaris*) and ginger (*Zingiber officinale*) at concentrations of 2.5–10 mg/mL; and chamomile (*Matricaria recutita*) and moringa (*Moringa oleifera*) at concentrations of 7.8–2.5 mg/mL [17,18,19,20].

Extracts from *Rosa* spp. are characterized by a high content of phenolic compounds, such as gallic acid, quercetin and anthocyanins, which possess both antimicrobial and antioxidant properties. As a result, they can be used in the food industry as natural preservatives, helping to inhibit pathogen growth and extend the shelf life of food products [21,22,23,24].

To ensure food safety without negatively affecting fermented food products, *Rosa* spp. extracts should be applied at concentrations that selectively inhibit the growth of undesirable microorganisms, while remaining harmless to the starter cultures responsible for fermentation. For instance, in the production of fermented dairy beverages or pickled products, low doses of the extract can be used to avoid interfering with lactic acid bacteria growth, while effectively inhibiting pathogens such as *Staphylococcus* spp. Furthermore, phenolic compounds can be metabolized by probiotic bacteria, thereby exhibiting prebiotic properties. It is also possible to design lactic acid bacteria–plant extract systems that demonstrate enhanced anti-*Staphylococcus* potential compared to either component used alone. Thus, *Rosa* spp. pseudo-fruit extract may serve as a natural alternative to synthetic preservatives, increasing food safety and meeting the growing consumer demand for products free from artificial additives [16,25,26].

The aim of this study was to assess the possibility of using extracts from *Rosa* spp. pseudo-fruits in limiting the growth of coagulase-negative *Staphylococcus* spp. bacteria isolated from food. The aim of this study was achieved by assessing changes in the degree of multiplication of bacterial biomass during a 72 h culture with the addition of *Rosa* spp. extracts and determining changes in the growth/death rate of the tested bacteria. In this study, based on the obtained results, a possible mechanism of the antagonistic effect of plant extracts on the tested *Staphylococcus* bacteria is proposed.

## 2. Results and Discussion

### 2.1. Growth Inhibition Zone of Test Bacteria Depending on the Extract Concentration

Table 1 presents the dependence of the size of the growth inhibition zone of coagulase-negative strains of bacteria from the genus *Staphylococcus* on the concentration of the tested extract. It was shown that the size of the growth inhibition zone of the tested bacteria from the genus *Staphylococcus* decreases with a decrease in the concentration of the extracts. Nevertheless, the observed dependence is not linear. A twice smaller growth inhibition zone of staphylococci was not observed with the next twice lower concentration of the extract.

For example, an extract from the whole pseudo-fruit of *Rosa canina* at a concentration of 400 mg/mL inhibited the growth of the reference strain *Staphylococcus aureus* ATCC 25923 at a level of 20 mm. In turn, in the case of a twice lower concentration of the extract, the growth inhibition zone was 18.33 mm, and at a four times lower concentration, it was equal to 16.33 mm. Thus, the obtained results of the size of growth inhibition zones between subsequent two-fold concentrations of extracts were not always statistically significant (within the same strain of the *Staphylococcus* spp.). A similar tendency was observed for the remaining strains.

It should also be emphasized that in the case of the last concentration limiting the growth of the tested bacteria of the *Staphylococcus* genus, which was defined as MIC, differences were also observed in the size of the growth inhibition zone of the tested bacteria. In most of the cases studied, the diameter of the growth inhibition zone at the MIC value was in the range of 11–14 mm, while in the case of the *Staphylococcus epidermidis* A5 strain, the growth inhibition zone at the MIC concentration for the extract obtained from the whole pseudo-fruit of *Rosa rugosa* was as much as 19 mm, while in the case of the extract from the flesh of *Rosa pomifera* ’Karpatia’ it was 18.5 mm.

In the case of the extract from the whole pseudo-fruit of *Rosa canina*, the highest sensitivity was observed in the case of the *Staphylococcus epidermidis* A5 strain and the reference strain *Staphylococcus aureus* ATCC 25923. In turn, the most resistant to the extract was the environmental isolate *Staphylococcus epidermidis* DSMZ 3270. The remaining tested bacterial strains showed similar sensitivity to the tested extract.

In the case of the extract from the flesh of *Rosa canina*, the highest sensitivity was noted in the case of the *Staphylococcus xylosus* M5 and *Staphylococcus capitis* KR6 strains (a comparable growth inhibition zone at an extract concentration of 200 mg/mL). The remaining tested strains showed similar resistance to the tested extract.

In the case of the extract from the whole pseudo-fruit of *Rosa rugosa*, it can be clearly observed that the highest resistance to the tested extract was demonstrated by the environmental isolate—*Staphylococcus haemolyticus* M6. For this strain, the extract at a concentration of 100 mg/mL had to be used to limit growth, while the other strains tested showed sensitivity to the extract at a concentration of 6.25–25 mg/mL. The extract obtained from the flesh of *Rosa rugosa* showed significantly lower antistaphylococcal activity compared to the extract from the whole pseudo-fruit. Most of the tested strains were resistant to the extract below a concentration of 400 mg/mL. The *Staphylococcus capitis* KR6 strain, on the other hand, showed quite high sensitivity to the extract obtained from the flesh of *Rosa rugosa*. However, the exception was the *Staphylococcus xylosus* M5 strain, in which case 12.5 mg/mL was the last concentration inhibiting growth.

It is also worth noting that in the case of this strain, the method of obtaining the extract is not important in the assessment of antistaphylococcal activity. A similar situation was observed in the case of the sensitivity of the *Staphylococcus xylosus* M5 strain to extracts obtained from *Rosa pomifera* ‘Karpatia’.

Our studies are consistent with the studies of Jafari-Sales et al. [27], in which the effect of methanol extracts from *Rosa damascena* on Gram-positive and Gram-negative bacteria was observed. In the studies of Jafari-Sales et al. [27] in the case of *Staphylococcus aureus*, it was observed that the diameter of the growth inhibition zone depended on the extract concentration, but this relationship was not linear. At an extract concentration of 400 mg/mL, it was 26 mm, while at a concentration of 50 mg/mL it oscillated around 20 mm. Thus, the MIC value of the tested extract was 6.25 mg/mL. The above authors showed that rose extracts exhibit antistaphylococcal activity, which is consistent with the results described in this paper. In turn, the studies by Jung et al. [28] showed that plant extracts obtained from 16 species of medicinal plants (including *Curcuma aromatica*, *Euphorbia humifusa*, *Glycyrrhiza uralensis*, *Lonicera japonica*, *Polygonum tinctorium*, *Sanguisorba officinalis* and *Scutellaria baicalensis*) exhibit antistaphylococcal activity. These studies showed that the antagonistic activity largely depended on the plant species. At a concentration of 40 mg/mL, high growth inhibition zones of the tested *Staphylococcus* bacteria were observed at a level of 10–25 mm. A two-fold reduction in the concentration of the extracts resulted in a reduction of growth inhibition zones to 9–15 mm, which is also consistent with our studies.

In the study by Safdar and Malik [29], aqueous extracts from *Rosa damascena* petals were analyzed for their effects on both Gram-positive and Gram-negative bacteria. For the *Staphylococcus aureus* strain, an inhibition zone of 35 mm was observed. Although this result is higher than the inhibition zones observed for the extracts tested in our study, it is important to note that Safdar and Malik [29] did not report the minimum inhibitory concentration (MIC) required to suppress bacterial growth. Their study only confirmed the potential use of rose extracts in limiting the growth of *Staphylococcus* species, without determining threshold concentrations such as the MIC value.

On the other hand, the study by Cendrowski et al. [30] examined the effects of whole pseudo-fruit extracts from *Rosa rugosa* on the growth of *Staphylococcus aureus* ATCC 25923 and *Staphylococcus epidermidis* ATCC 12228. The minimum inhibitory concentration of the extracts ranged from 16 mg/mL to over 128 mg/mL, depending on the extraction method used. Therefore, the *Rosa* spp. extracts examined in our study demonstrated, in most cases, a higher antistaphylococcus potential—especially when considering the same rose species (*Rosa rugosa*).

Analyzing the coefficient of determination R^2^ in the evaluation of the antagonistic activity of the tested extracts (Table 2), it was found that the antagonistic activity of the tested extracts, although dependent on the extract concentration, is not linear. In 17 of the cases studied, there was not enough data to determine the coefficient of determination R^2^ (marked X in the table). Since there is no linear relationship when assessing antagonistic activity, it was decided to determine the coefficient of determination using a logarithmic trend line.

The determined value of R^2^ allowed us to notice that in all the cases studied, this fitting model was more justified (a higher coefficient of determination than in the linear fitting). Thus, the coefficient of determination in the linear model did not exceed the value of 0.8 (in 15 out of 25 cases, it was lower than 0.5), while in the logarithmic model, in none of the cases studied was it lower than 0.6 (in seven cases studied, it was higher than 0.8).

To predict the effectiveness of compounds that inhibit microbial growth and to understand the behavior of a given test strain, the mathematical modeling of microbial responses is often used. The validity of the applied model is indicated by its good fit to the experimental data, expressed by the R^2^ coefficient. This coefficient ranges from 0 to 1, with values closer to 1 indicating a better fit of the model to the observed data [31].

A different trend was observed in the studies by Bshabshe et al. [32], in which the antagonistic activity of mango seed extracts (towards *Staphylococcus aureus* strains) was tested. When using extract concentrations in the range of 0.05–050 mg/mL (subsequent 10-fold dilutions), the antagonistic activity of the tested extract represented a linear fit, in which the R^2^ coefficient was 0.9841. In the case of the rose extracts we tested, such a high R^2^ coefficient value was recorded only in the case of the *Staphylococcus xylosus* M5 strain culture with the addition of *Rosa canina* flesh extract.

However, it should be noted that there is a positive correlation between the extract concentration and the size of the growth inhibition zone, although it does not represent a linear relationship. A similar trend was observed by Beressa et al. [33], who studied the antifungal activity of *Echinops kebericho* Mesfin essential oil against *Candida* fungi. Thus, the R^2^ coefficient value ranged from 0.4115 to 0.6452 (depending on the species tested), which corresponds to the R^2^ coefficient value defining the antagonistic activity of rose extracts.

### 2.2. Growth Dynamics of the Tested Strains of the Staphylococcus Genus in Cultures with the Addition of Extracts from Rosa spp.

**Figure 1:**
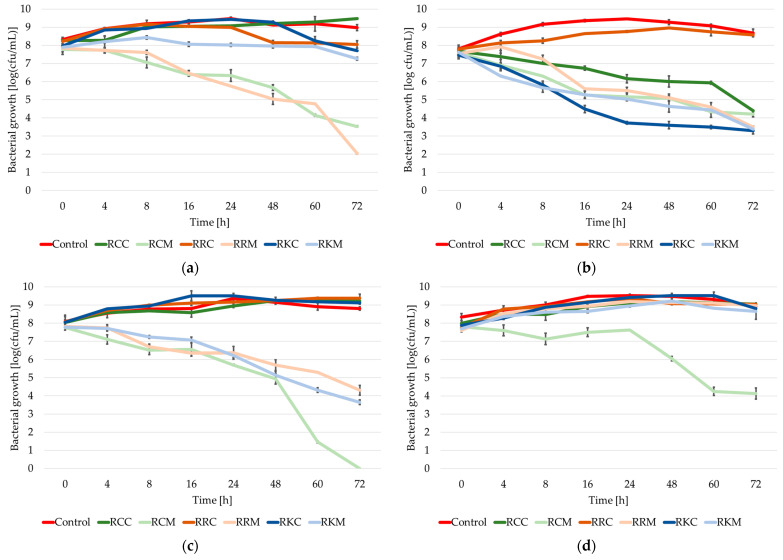

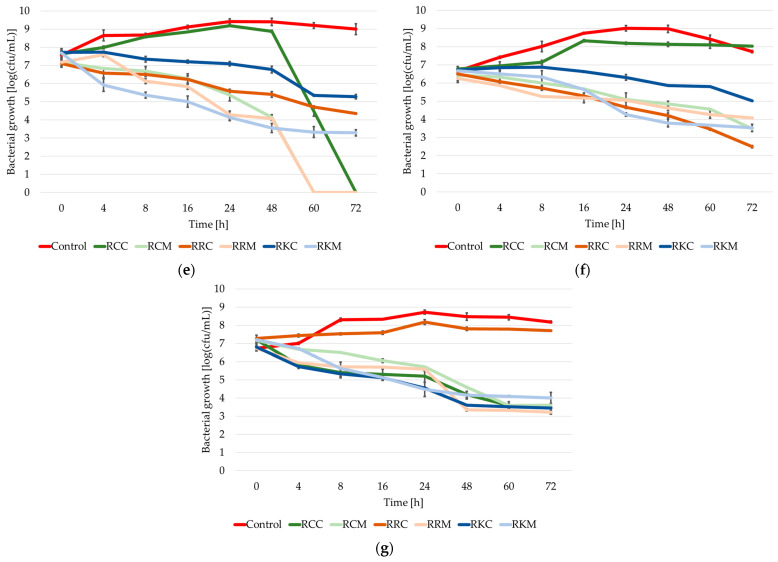
Change in the growth dynamics of the strain in cultures with the addition of extracts from *Rosa* spp.: (**a**) *Staphylococcus aureus* ATCC 25923; (**b**) *Staphylococcus epidermidis* DSMZ 3270; (**c**) *Staphylococcus epidermidis* A5; (**d**) *Staphylococcus xylosus* M5; (**e**) *Staphylococcus haemolyticus* M6; (**f**) *Staphylococcus capitis* KR6; (**g**) *Staphylococcus warneri* KR2A. Tested cultures: control—culture without the addition of extracts; RCC—cultivation with the addition of extract from the whole pseudo-fruit of *Rosa canina*; RCM—cultivation with the addition of *Rosa canina* flesh extract; RRC—cultivation with the addition of extract from the whole pseudo-fruit of *Rosa rugosa*; RRM—cultivation with the addition of *Rosa rugosa* flesh extract; RKC—cultivation with the addition of extract from the whole pseudo-fruit of *Rosa pomifera* ‘Karpatia’; RKM—cultivation with the addition of *Rosa pomifera* ‘Karpatia’ flesh extract.

When analyzing the growth course of the tested strains of the *Staphylococcus* genus in control cultures (without the addition of extracts), in most of the tested cases during the first 24 h of incubation, a logarithmic increase in biomass was observed, ending with the achievement of the maximum level of multiplication (the maximum growth was from 8.72 logarithmic units (in the case of the *Staphylococcus warneri* KR2A strain) to 9.52 logarithmic units (in the case of the *Staphylococcus xylosus* M5 strain)) (Figure 1).

Thus, the degree of biomass multiplication in the control cultures after 24 h of incubation was 14–16% higher than the initial degree of biomass multiplication in the case of the strains *Staphylococcus aureus* ATCC 25923, *Staphylococcus xylosus* M5 and *Staphylococcus epidermidis* A5; 21–24% higher than the initial degree of biomass multiplication in the case of the strains *Staphylococcus epidermidis* DSMZ 3270 and *Staphylococcus haemolyticus* M6; and 30–35% higher than the initial degree of biomass multiplication in the case of the isolates cultured (*Staphylococcus capitis* KR6 and *Staphylococcus warneri* KR2A).

In the following hours of incubation, the number of cells of the tested strains remained in the stationary phase and/or passed into the phase of mild dying. In most of the cases studied, the difference between the maximum and final degree of biomass multiplication was in the range of 0.4–0.8 logarithmic units, the only exception being the *Staphylococcus capitis* KR6 strain, in which case the difference in the degree of biomass multiplication (between the maximum degree of biomass multiplication and the final degree of biomass multiplication) was observed at the level of 1.28 logarithmic units.

In all the cultures studied, the final degree of cell multiplication was higher than the initial degree of multiplication (usually by 0.6–1.0 logarithmic units; the exceptions were the *Staphylococcus haemolyticus* M6 and *Staphylococcus warneri* KR2A strains, in which case this difference oscillated at around 1.45 logarithmic units).

In cultures with the addition of the extract from the whole pseudo-fruit of *Rosa canina*, it can be observed that in the case of the strains *Staphylococcus aureus* ATCC 25923, *Staphylococcus epidermidis* A5 and *Staphylococcus xylosus* M5, a gradual increase in cells occurs, as a result of which, after 72 h incubation, the degree of bacterial multiplication is 1–1.3 logarithmic units higher. A similar tendency was observed in the case of the *Staphylococcus haemolyticus* M6 strain up to 48 h of incubation. After this time, the culture passed into an intensive phase of dying out, ending with the complete inability of the tested strain to grow.

In the case of the *Staphylococcus capitis* KR6 strain, an increase in the degree of cell multiplication was observed up to 16 h of incubation, after which the strain passed into the stationary phase. In the case of the strains *Staphylococcus epidermidis* DSMZ 3270 and *Staphylococcus warneri* KR2A, from the beginning in the culture with the addition of the extract from the whole *Rosa canina* pseudo-fruit at a concentration of ¼ MIC, a gradual decrease in the degree of bacterial multiplication was observed, which after 72 h was between 3 and 4 logarithmic orders.

In the case of cultures with the addition of *Rosa canina* flesh extract at a concentration of ¼ MIC for each of the tested strains, a gradual decrease in the degree of cell multiplication was observed. The decrease in the degree of biomass multiplication ranged from 2.95 logarithmic orders in the case of the *Staphylococcus capitis* KR6 strain to 4.24 logarithmic orders in the case of the *Staphylococcus aureus* ATCC 25923 strain. In the case of the *Staphylococcus epidermidis* A5 and *Staphylococcus haemolyticus* M6 strains, complete inhibition of growth occurred during 60–72 h of culture.

In cultures with the addition of the extract from the whole pseudo-fruit of *Rosa rugosa*, a dualistic response of strains of the *Staphylococcus* genus was observed. On the one hand, in the cultures of the strains *Staphylococcus epidermidis* DSMZ 3270, *Staphylococcus epidermidis* A5, *Staphylococcus xylosus* M5 and *Staphylococcus warneri* KR2A, an increase in cells was observed during 72 h incubation (no more than 17%); on the other hand, in the case of the strains *Staphylococcus aureus* ATCC 25923, *Staphylococcus haemolyticus* M6 and *Staphylococcus capitis* KR6, an antagonistic effect of the tested extract was noted. Thus, the degree of cell multiplication was lower by 0.17 logarithmic orders in the case of *Staphylococcus aureus* ATCC 25923, by 2.76 logarithmic orders in the case of *Staphylococcus haemolyticus* M6 and by as much as 4 logarithmic orders in the case of *Staphylococcus capitis* KR6.

In the cultures with the addition of *Rosa rugosa* flesh extract, only the *Staphylococcus xylosus* M5 strain was observed to be able to grow at an extract concentration of ¼ MIC. The degree of cell multiplication after 72 h incubation was 1.35 logarithmic orders higher than the density of the introduced inoculum. In the remaining cases studied, a decrease in the degree of cell multiplication was noted, ranging from 2 to almost 6 logarithmic orders. In the case of the *Staphylococcus haemolyticus* M6 strain, complete inhibition of growth was noted after 60 h of incubation (a decrease in the degree of multiplication of bacterial cells above 7 logarithmic orders).

The addition of the extract from the whole pseudo-fruit of *Rosa pomifera* ‘Karpatia’ to the culture of the strains *Staphylococcus aureus* ATCC 25923, *Staphylococcus epidermidis* A5 and *Staphylococcus xylosus* M5 at a concentration of ¼ MIC did not show any activity limiting the growth of the tested staphylococci up to 48 h of incubation. Thus, after 72 h of incubation, the degree of multiplication of the tested bacterial cells in the culture with the addition of the extract was 0.9–1.1 logarithmic units higher than the introduced inoculum. On the other hand, the *Staphylococcus aureus* ATCC 25923 strain in the culture with the extract added entered a mild die-off phase after 48 h of incubation, as a result of which after 72 h the degree of cell multiplication in the culture with the extract was 0.26 logarithmic units lower than the initial point.

In the remaining cases studied, antistaphylococcal activity of the extract from the whole pseudo-fruit of *Rosa pomifera* ‘Karpatia’ was observed, though it varied depending on the tested strain. Significantly lower antagonistic activity was noted against *Staphylococcus haemolyticus* M6 and *Staphylococcus capitis* KR6 compared to *Staphylococcus epidermidis* DSMZ 3270 and *Staphylococcus warneri* KR2A. Similarly to the culture with the addition of the extract from the flesh of *Rosa rugosa*, the extract from the flesh of *Rosa pomifera* ‘Karpatia’ did not significantly affect the *Staphylococcus xylosus* M5 strain in a way that limited bacterial growth. After 96 h of incubation, the degree of biomass multiplication was almost 1 logarithmic unit higher than that of the inoculum. The extract also showed low antagonistic activity against *Staphylococcus aureus* ATCC 25923. In the remaining cases studied, a decrease in the degree of biomass multiplication by 3–4.5 logarithmic units was observed from the beginning of incubation.

Analyzing all the obtained results, it is not possible to clearly determine in which culture the degree of cell multiplication after 72 h incubation was the highest. Although in most cases, the highest bacterial cell count was observed in the control culture, the *Staphylococcus xylosus* M5 strain exhibited comparable levels of multiplication across nearly all the tested conditions. The only exception was the culture with the addition of *Rosa canina* flesh extract, where after 72 h, bacterial multiplication was 4.79 logarithmic units (54%) lower than in the cultures with other extracts.

For the *Staphylococcus epidermidis* A5 strain, the highest degree of bacterial multiplication after 72 h was observed not only in the control culture but also in the one with the addition of pseudo-fruit extracts (with differences of 4–7% between samples). In the *Staphylococcus aureus* ATCC 25923 and *Staphylococcus capitis* KR6 strains, the highest degree of bacterial multiplication at the 72 h mark was found in cultures with the addition of the extract from the whole *Rosa canina* pseudo-fruit. In contrast, for the *Staphylococcus epidermidis* DSMZ 3270 strain, the highest degree of cell multiplication was observed both in the control culture and in the culture with the extract from the whole *Rosa rugosa* pseudo-fruit.

It is also difficult to clearly determine in which tested variant the degree of cell multiplication was the lowest. In the *Staphylococcus aureus* ATCC 25923 strain, the lowest bacterial multiplication was observed in cultures with the addition of *Rosa rugosa* flesh extract, where bacterial multiplication was almost 80% lower than in the control culture. For *Staphylococcus epidermidis* A5 and *Staphylococcus xylosus* M5, the lowest cell multiplication was observed in cultures with the addition of *Rosa canina* flesh extract. In the *Staphylococcus xylosus* M5 strain, multiplication was more than 50% lower than in the control culture, whereas for *Staphylococcus epidermidis* A5, no bacterial growth was observed after 72 h.

A similar trend was observed in *Staphylococcus haemolyticus* M6, where the addition of *Rosa rugosa* flesh extract and both *Rosa canina* extracts led to a complete (100%) reduction in bacterial multiplication after 72 h of incubation. In the *Staphylococcus capitis* KR6 strain, the lowest degree of biomass multiplication was observed in the culture with the addition of the extract from the whole *Rosa rugosa* pseudo-fruit, with bacterial multiplication reduced by almost 70% compared to the control culture.

However, it is most difficult to determine the lowest degree of bacterial multiplication in *Staphylococcus epidermidis* DSMZ 3270 and *Staphylococcus warneri* KR2A. In *Staphylococcus epidermidis* DSMZ 3270, cultures with the addition of extracts from *Rosa pomifera* ‘Karpatia’ and *Rosa rugosa* flesh exhibited 59–62% lower multiplication rates compared to the control. In *Staphylococcus warneri* KR2A, the degree of biomass multiplication after 72 h of incubation was reduced by 50–60% in nearly all cultures with extracts, except for the culture with the addition of the extract from the whole pseudo-fruit of *Rosa rugosa*.

In summary, it can be stated that extracts from *Rosa* spp. added to the culture of bacteria of the *Staphylococcus* genus can both stimulate and limit the growth of staphylococci. First of all, they affect the extension of the adaptation phase—which is particularly visible in the case of the *Staphylococcus aureus* ATCC 25923 strain. The extended adaptation phase can end either with immediate entry into the dying phase, which, depending on the culture tested, can be mild or severe, or with the logarithmic growth phase, which can occur in one or two stages. Similar results were observed by Liu et al. [34], who studied the effect of ethanol extracts from freeze-dried *Pingyin* rose buds on the growth of the *Staphylococcus aureus* ATCC 25923 strain. In the case of the extract concentration at a level of ¼ MIC, the the authors of the study observed a lower ability to grow than in the control sample. The tested strain showed a growth capacity of no more than one logarithmic unit during the first 5 h of incubation. The level of cell multiplication remained unchanged until the 24th hour of culture. Thus, in the case of the control culture, an increase in the degree of staphylococci multiplication was observed from 5 logarithmic orders to almost 9 logarithmic orders.

Zeng et al. [35], after determining the MIC value of the aqueous extract of *Polygonum chinense* L. in relation to *Staphylococcus aureus*, also examined the effect of different concentrations of the extract on the growth dynamics. During the 24 h incubation, they did not observe significant changes in the growth dynamics of the tested strain between the control culture without the addition of the extract and the one to which the extract was added at a concentration of ¼ MIC. Although the initial number of microorganisms in the culture with the addition of the extract was as much as 1 logarithmic unit lower than in the control culture, within 10 h the degree of bacterial cell multiplication in both cultures equalized and remained the same until 24 h. In turn, cranberry extract rich in anthocyanins showed the ability to inhibit eight strains of *Staphylococcus aureus* (cultures with the addition of the extract at a concentration of ½ MIC—concentration of 5 mg/mL). Interestingly, after 7 h of incubation, the presence of *Staphylococcus aureus* bacterial cells was not determined in the culture with the addition of the extract [36]. This proves that it is possible to completely inhibit the growth of *Staphylococcus* bacteria in the culture with the addition of plant extracts, which is consistent with our studies. Such a situation was noted in the case of the *Staphylococcus epidermidis* A5 strain culture with the addition of *Rosa canina* flesh extract and the *Staphylococcus haemolyticus* M6 strain culture with the addition of *Rosa canina* and *Rosa rugosa* flesh extracts.

The rose extracts tested limited the growth of environmental, coagulase-negative bacteria of the *Staphylococcus* genus at a concentration of 3.125–500 mg/mL. However, in the study of changes in growth dynamics, it was decided to use a concentration equal to ¼ MIC (0.781–125 mg/mL). Therefore, it is worth noting that at such low concentrations, a limitation in the growth of the tested strains was observed in most of the cases studied. For example, Guo et al. [37], in which the ethanol extract of *Amaranthus tricolor* showed antistaphylococcal activity at a concentration of 80–320 mg/mL against seven strains of *Staphylococcus aureus*. Thus, the diameter of the growth inhibition zones at the MIC value was in the range of 12–13 mm. An analysis of the growth curve of the tested strains at a concentration of ½ MIC showed that the extract slightly limited the growth of the tested microorganisms. The degree of bacterial cell multiplication was comparable to the control culture during a 24 h culture.

The tested *Rosa* spp. extracts exhibited high antistaphylococcus activity, leading to a significant reduction in biomass proliferation in cultures containing these extracts. However, not all plant extracts are capable of completely inhibiting bacterial growth in co-culture conditions. For example, in the study by Kitsiou et al. [38], grape seed extract reduced the biomass proliferation of *Listeria monocytogenes* 10403S WT by only 2.9 logarithmic units during 24 h incubation.

Nevertheless, the microbial response to the presence of phenolic compounds in the growth environment is highly dependent on the tested strain, as well as the plant used for extract preparation. According to the study by Gavrill et al. [39], extracts from oregano, thyme and basil were able to nearly completely inhibit the growth of *Salmonella* Typhimurium.

The influence on changes in the growth dynamics of *Staphylococcus* bacteria may be primarily exerted by ellagitannins present in plant extracts. According to studies conducted by Puljula et al. [20], salicarinin A and rugosin D completely inhibited the growth of *Staphylococcus aureus* at a concentration of 0.5 mM. On the other hand, the use of compounds such as casuarictin, tellimagrandins I and II, pentagalloylglucose, stachyurin, casuarinin, vescalagin, castalagin, rugosin E, sanguiin H-6 and lambertianin C also effectively inhibited the growth of the tested bacteria during 24 h culture. A decrease in the multiplication of biomass from 10^9^ cfu/mL to 10^8^–10^2^ cfu/mL was observed. Thus, Puljula et al. [40] showed that rugosin was the compound that had the greatest impact on limiting the growth of *Staphylococcus* bacteria. Following this thesis, it should be emphasized that the rose extracts tested in this work contained from 28 to 36 g of phenolic compounds in 100 g of lyophilisate [41]. Ellagitannins in the extracts of *Rosa rugosa* constituted 21–35% of all the phenolic compounds tested, whereas in the case of *Rosa canina* and *Rosa pomifera* ‘Karpatia’ it was only 3–9%, which may confirm the above statement.

Previous studies on plant extracts have shown that the main groups of phenolic compounds identified in their composition are flavanols and ellagitannins. The extracts obtained from *Rosa rugosa* contained significantly higher amounts of ellagitannins—nearly five to eight times higher than in other extracts. Regardless of the *Rosa* spp. variety, extracts from the pulp had a lower ellagitannin content compared to extracts from whole pseudo-fruits. The opposite trend was observed for flavanols [41].

In the study by Milala et al. [41] (which represents the first stage of the research presented above), a correlation between the main polyphenol groups in the extracts and their antistaphylococcus potential was also demonstrated (at an extract concentration of 500 mg/mL). For extracts obtained from whole pseudo-fruits, the antistaphylococcus potential was positively correlated with ellagitannin content in most cases. However, for flesh extracts, a strong positive correlation with ellagitannin content was observed only for *Staphylococcus epidermidis* DSMZ 3270 and *Staphylococcus xylosus* M5.

Additionally, it was observed that the presence of flavanols in the tested extracts did not have a significant inhibitory effect on the growth of the studied bacterial strains. This finding aligns with the results obtained by Puljula et al. [40] and Puupponen-Pimia et al. [42].

### 2.3. The Growth/Death Rate of Staphylococcus spp. Bacteria in Cultures with the Addition of Extracts

The minimum inhibitory concentration (MIC) and minimum bactericidal concentration (MBC) are the most commonly used parameters to determine the antimicrobial activity of plant extracts. It is worth noting that time-based evaluation (observing bacterial growth or death over time) at different concentrations of an antimicrobial compound allows for a more comprehensive and precise characterization of its antimicrobial effectiveness [43].

Table 3 presents the value of the ∆μ coefficient—informing one about the growth or death rate of the tested microorganisms. In the case of the control culture without the addition of *Rosa* spp. extracts, it was observed that all seven tested strains showed the ability to grow in the first 24 h of incubation. The growth rate coefficient of the strains was in the range of 0.006–0.015 h^−1^. In turn, extending the culture time by another 48 h resulted in the culture of the tested strains entering the death phase, with a death rate of 0.001–0.003 h^−1^.

In the cultures with the addition of the extract obtained from the whole pseudo-fruit of *Rosa canina*, the ability to survive was observed in as many as five out of seven tested cases during the first 24 h of incubation. The growth rate coefficient of the tested strains was in the range of 0.004–0.009 h^−1^. In the case of the *Staphylococcus epidermidis* DSMZ 3270 and *Staphylococcus warneri* KR2A strains, a gradual decrease in the biomass multiplication rate was observed, translating into a die-off rate of 0.009–0.012 h^−1^. In the case of both strains, extending the culture to 72 h still resulted in a decrease in the biomass multiplication rate, with a lower die-off rate than during the first 24 h of incubation. Moreover, the *Staphylococcus haemolyticus* M6 strain also entered the phase of acute die-off (a die-off rate coefficient of 0.021 h^−1^). In the case of the remaining strains tested, no significant change in the growth/die-off rate of the biomass multiplication rate was observed.

This is particularly significant in comparison with the cultures of the tested *Staphylococcus* bacteria with the addition of *Rosa canina* flesh extract. In all seven tested cases, a decrease in the degree of biomass multiplication was observed, with a die-off rate coefficient of 0.001–0.011 h^−1^—depending on the tested strain. The lowest die-off rate was noted for the *Staphylococcus xylosus* M5 strain. For the remaining six tested strains, it was higher than 0.008 h^−1^. However, over the next 48 h, a gradual decrease in the degrees of biomass multiplication of the tested strains was still observed. The die-off rate was higher than in the first 24 h for the following strains: *Staphylococcus aureus* ATTC 25923, *Staphylococcus epidermidis* A5, *Staphylococcus xylosus* M5 and *Staphylococcus haemolyticus* M6. This indicates that after 24 h in the cultures with the addition of the extract, a phase of dying was noted with a much more rapid course than in the first 24 h.

In the case of the cultures with the addition of the extract from the whole pseudo-fruit of *Rosa rugosa*, only in two studied cases (*Staphylococcus haemolyticus* M6, *Staphylococcus capitis* KR6) in the first 24 h was a decrease in the degree of biomass multiplication observed, translating into a dying rate of 0.009–0.012 h^−1^. In the remaining studied cultures, the growth rate was in the range of 0.004–0.009 h^−1^. The extension of the incubation time to 72 h allowed us to observe that between the 24th and 72nd hour of culture, in most of the studied cases, a slight die-off of the culture was observed (the growth rate did not exceed 0.001 h^−1^, while the die-off rate did not exceed 0.002 h^−1^). The exceptions were the cultures of the *Staphylococcus haemolyticus* M6 and *Staphylococcus capitis* KR6 strains, in which cases intensive die-off was still observed, expressed by a die-off rate coefficient at a level of 0.005–0.01 h^−1^.

In the case of cultures with the addition of *Rosa rugosa* flesh extract, it can be observed that already in the first 24 h of incubation, most of the studied strains went through the intensive die-off phase. The die-off rate coefficient was within the range of 0.008–0.017 h^−1^. Only in the case of the *Staphylococcus xylosus* M5 strain was a gradual increase in biomass noted, with a growth rate of 0.009 h^−1^. On the other hand, the extension of the culture time resulted in a gradual decrease in the degree of biomass multiplication, with a die-off rate of 0.004–0.021 h^−1^ depending on the strain tested.

In the case of cultures with the addition of the extract from the whole fruit of *Rosa pomifera* ‘Karpatia’, it can be observed that three of the tested strains (*Staphylococcus aureus* ATCC, *Staphylococcus epidermidis* A5 and *Staphylococcus xylosus* M5) retained the ability to grow, with a growth rate of 0.008 h^−1^ during the first 24 h. The remaining tested strains entered the die-off phase with a varied course. The most intensive die-off was noted for the *Staphylococcus epidermidis* DSMZ 3270 strain (a die-off rate coefficient 0.021 h^−1^) and *Staphylococcus warneri* KR2A (a die-off rate coefficient 0.014 h^−1^). In the case of the *Staphylococcus haemolyticus* M6 and *Staphylococcus capitis* KR6 strains, the die-off rate was 0.003 h^−1^. Extending the culture time for all the strains tested resulted in entering the mild die-off phase. The die-off rate coefficient was 0.001–0.05 h^−1^.

A similar trend was observed in cultures with the addition of *Rosa pomifera* ‘Karpatia’ flesh extract. In most of the strains tested, a gradual decrease in the degree of biomass multiplication was already noted from the first hours of culture. The die-off rate coefficient ranged from 0.007 h^−1^ to 0.019 h^−1^. The only exception was the *Staphylococcus aureus* ATCC 25923 strain, in which a slight change in the degree of biomass multiplication was observed (a growth rate coefficient at the level of 0.001 h^−1^). In turn, between 24 and 72 h of incubation, all the cultures entered the die-off phase (a die-off rate coefficient at the level of 0.001–0.009 h^−1^).

In summary, in most of the studied cases a negative value of the growth rate was observed, which indicates a decrease in the degree of bacterial cell multiplication in cultures with the addition of extracts (die-off rate coefficient). Similar results were obtained by Silva et al. [44], who studied the effect of mint, lemon balm and sage extracts on the survival of *Staphylococcus aureus* in ripening cheeses. They showed that the use of spearmint extract, compared to the control group, allowed for determining a lower growth rate, which suggests a higher rate of *Staphylococcus* death. Thus, spearmint extract helped to shorten the time of *Staphylococcus aureus* dying out during the cheese-ripening process. The results of the study obtained by Silva et al. [44] also indicate that sometimes plant extracts can increase the growth rate of *Staphylococcus* spp., which was observed in the case of lemon balm or sage extract (a higher growth rate compared to the control sample). The use of fruit rose extracts significantly shortens the time needed to inhibit the growth of bacteria from the *Staphylococcus* genus.

According to the study conducted by Silva et al. [44], plant extracts shortened the time needed for the *Staphylococcus aureus* culture to pass into the dying-out phase. Although a reduction in the degree of biomass multiplication was observed in the control culture, it was not as pronounced as in the cultures with the addition of extracts, which is identical to the results described in our work.

A similar trend was observed in the study by Buldain et al. [45]. Using essential oils from *Melaleuca armillaris* and applying the Gompertz model, a reduction in the growth rate (μ) and biomass proliferation of *Staphylococcus aureus* was noted at an essential oil concentration of 0.5 MIC, compared to the control culture.

### 2.4. Scanning Electron Microscopy—Assessment of Changes in Staphylococcus spp. Cell Morphology

Scanning electron microscopy is one of the techniques that allows the imaging of surface microstructures. The idea of scanning electron microscopy is primarily based on scanning the surface of the sample with a nanometer beam of electrons formed by an electro-optical microscope system [46].

**Figure 2:**
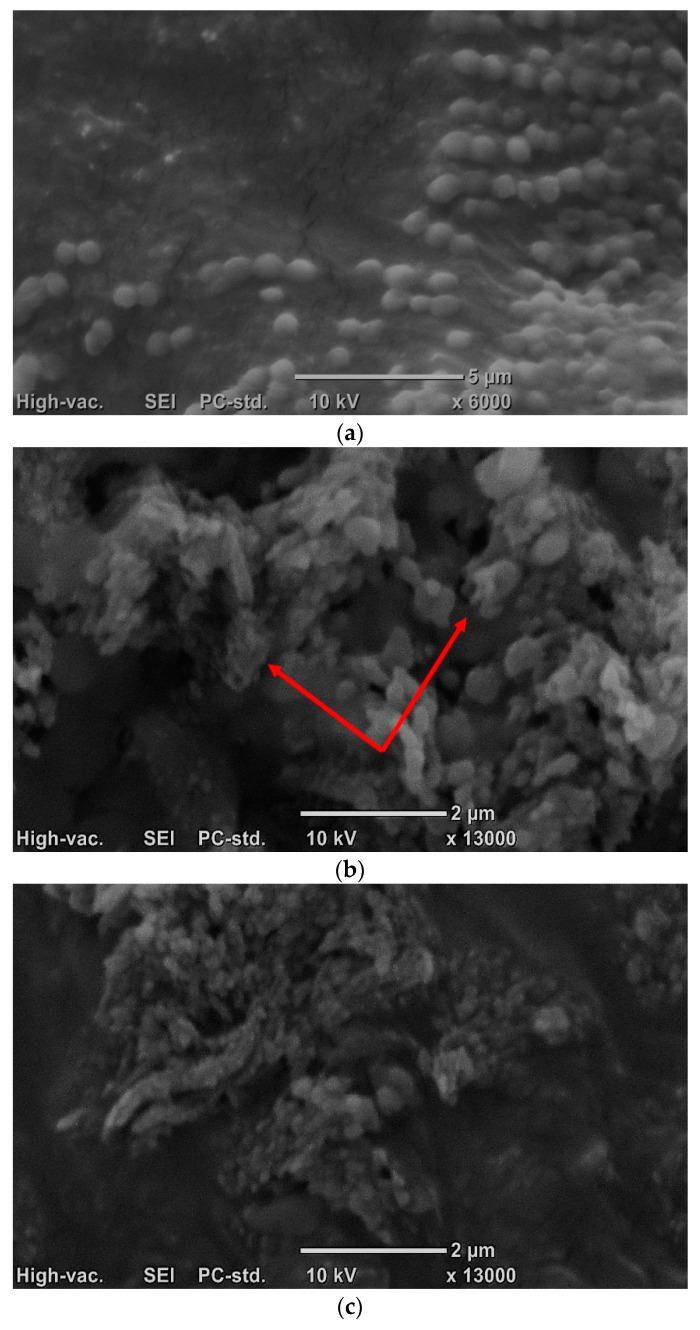
SEM analysis: (**a**) 24 h culture of *Staphylococcus xylosus* M5 bacteria (optimal conditions for the growth of the tested strain; Nutrient Broth liquid culture, 37 °C); (**b**) 24 h culture of the *Staphylococcus xylosus* M5 strain (Nutrient Broth, 37 °C) with the addition of freeze-dried *Rosa canina* flesh extract at a concentration of ¼ MIC (the red arrow marks the cells of *Staphylococcus* bacteria); (**c**) freeze-dried extract of *Rosa canina* flesh dissolved in 5% DMSO.

Figure 2 shows morphological changes in *Staphylococcus* bacteria cells in a culture with the addition of extracts from fruit roses, using the example of a culture of the *Staphylococcus xylosus* M5 strain and an extract from the flesh of *Rosa canina*. In the case of *Staphylococcus xylosus* M5 grown in conditions optimal for the growth of the strain (control sample), it can be observed that *Staphylococcus* cells are light, round and smooth (Figure 2a). The cells shown in the figure have a diameter of 0.5–1 μm. They form irregular groups characteristic of bacteria from the *Staphylococcus* genus. The cell walls remained clean, uniform and intact. Only in the case of very few units were there slight breakdowns in the cell wall structure (cells slightly collapsed, resembling erythrocytes), which is characteristic of older cells introduced with the inoculum.

As a result of the action of *Rosa* spp. extracts on *Staphylococcus* spp. bacterial cells, almost all the edges of the *Staphylococcus* cell walls become wrinkled (Figure 2b). The cocci change their morphology; they become deformed. The boundaries between cells become irregular, indicating a lack of integrity in the external membranes and cell walls. Most of them are ellipsoidal. Some of the cells are much smaller (in the case of ellipsoidal cells, up to 0.3 µm in width and up to 0.5 µm in length; in the case of spherical cells, the diameter is 0.2–0.4 µm), which may be related to the fact that the compounds contained in the extracts bind with components of the cell walls—leading to changes in cell membrane permeability and subsequent cell lysis.

It should also be noted that in the microscopic image of the culture with the addition of extracts, significantly fewer cells were observed than in the control culture. Additionally, phenolic compounds contained in rose extracts can combine with proteins in the cell walls. In this way, they ‘stick’ to the cell and hinder its proper growth (red arrow). Typical cell–extract complexes appear as irregular structures with dimensions of 0.5–1 µm. These complexes can also contain fragments of damaged cells.

A similar trend was observed by Morshady et al. [47], who reported that *Staphylococcus aureus* cells exposed to natural phenolic compounds (mainly hydroquinones) undergo significant deformation. Morshady et al. [47] also observed the formation of aggregates associated with fragmentation and the clustering of damaged cell debris. Cells treated with phenolic compounds exhibited an uneven size and shape, as well as an irregular arrangement.

Guo et al. [37] similarly observed changes in the morphology of *Staphylococcus* spp. cells under the influence of olive polyphenolic extract at MIC and 2MIC concentrations after 4 h of incubation. These changes were likely caused by the leakage of cytoplasm and intracellular components.

Zeng et al. [35] demonstrated that an aqueous extract of *Polygonum chinense* L. also induced morphological changes in *Staphylococcus aureus* cells. After 20 h of incubation, vesicles were visible on the surface of the cell walls. In addition, the cells were arranged irregularly and exhibited distortion. Contrary to the previously cited studies, Zeng et al. [35] observed an increase in cell volume.

According to the study by Chen et al. [48], a 3 h exposure to phenolic compounds contained in tea (previously oxidized using polyphenol oxidase and peroxidase) resulted in *Staphylococcus aureus* cells appearing more wrinkled than in the control culture. Characteristic dents were also observed. Additionally, growth curve analysis showed that in the test sample (with the addition of polyphenols), the number of cells expressed in OD (λ = 600 nm) was lower than in the control sample. Although an increase in absorbance was observed during the 7 h culture in the samples with the addition of phenolic compounds, the biomass level from the second hour of incubation onward was always statistically significantly lower than in the sample without these compounds.

## 3. Materials and Methods

### 3.1. Biological Material

The biological material in this work consisted of five strains isolated from food, whose species affiliation was confirmed using genetic methods (16S rRNA sequence analysis). The nucleotide sequences of selected isolates were deposited in the NCBI database (GenBank). The strains selected for the study came from fresh, unpasteurized cow’s milk: *Staphylococcus xylosus* M5 strain (GenBank deposit number: MW 776359) and *Staphylococcus haemolyticus* M6 strain (MW 776358); radish sprouts (country of origin: Poland): *Staphylococcus capitis* KR6 (MW 776357) and *Staphylococcus warneri* KR2A (MW 776360); as well as a dietary supplement (powdered acai berry extract—*Euterpe oleracea*, manufacturer A-Z Medica, (Gdańsk, Poland): *Staphylococcus epidermidis* A5 strain (MW 040699). At earlier stages of work, these strains were classified as coagulase-negative and multidrug-resistant [49].

The control sample consisted of two reference strains: *Staphylococcus aureus* ATCC 25923 and *Staphylococcus epidermidis* DSMZ 3270.

### 3.2. Studied Material

The research material consisted of six extracts obtained from three popular rose species in Poland: *Rosa canina*, *Rosa rugosa* and *Rosa pomifera* ‘Karpatia’. Pseudo-fruits for the preparation of extracts were obtained from the Institute of Horticulture, the State Research Institute in Skierniewice; they were collected at the end of September 2018. Extracts were prepared from both the flesh (which is the fleshy cover of the true fruit; muscular walls, which after ripening take on a color from orange to purple, sometimes black) and the whole pseudo-fruit (apparent fruit, i.e., the expanded flower base–hypanthium; commonly called the whole fruit; the whole pseudo-fruit consists of a fleshy cover—flesh—and hard achenes—the true fruit) of the above-mentioned rose varieties. The method of preparation and characteristics of the extracts are described in Milala et al. [41]. Plant extracts were obtained from the whole pseudo-fruits of three rose species: *Rosa canina*, *Rosa rugosa* and *Rosa pomifera* ‘Karpatia’. The plant material was ground using liquid nitrogen and a multifunctional machine (SPOMASZ-Nakło, Nakło, Poland), followed by liquid extraction using acetone/water/formic acid mixtures of 60:39.95:0.05 [*v/v/v*]. The process included dynamic extraction (8 h, 135 rpm) and static extraction (16 h), lasting a total of 24 h. The obtained extracts were decanted, combined and concentrated using a vacuum evaporator. Subsequently, extract purification was performed using a chromatographic column. Adsorbed polyphenols were eluted with ethanol/water/formic acid mixtures of 10:89.99:0.01 [*v/v/v*] and 60:39.99:0.01 [*v/v/v*]. After further concentration in a vacuum evaporator, the final step involved freeze-drying the extracts.

The characterization of the extracts included determining their flavanol and procyanidin content using the HPLC-FD method, as well as qualitative and quantitative analysis of selected polyphenols using UHPLC-DAD-MS. The first stage involved fluoroglucinolysis, followed by HPLC chromatographic analysis with fluorescence detection, allowing for the identification and quantification of flavan-3-ols. In the second stage, UHPLC coupled with a diode array detector (DAD) and a mass spectrometer (MS) was used to identify and quantify ellagitannins, ellagic acid and flavonols based on a comparison with standards and literature data [41].

### 3.3. Antimicrobial Properties of Extracts

In order to assess the antistaphylococcal properties of the rose extracts, from a 24 h culture of *Staphylococcus* bacteria (10^8^–10^9^ cfu/mL), 1 mL of suspension was taken and inoculated onto Nutrient Agar (Merck, Darmstadt, Germany). After solidifying the agar, six wells were cut in the plate using a sterile cork borer (Ø = 10 mm). Then, 100 µL of appropriately diluted extracts was introduced into the wells. The study utilized extract concentrations of 500, 400, 300, 200, 100, 50, 25, 12.5, 6.25, 3.13 and 1.56 mg of extract lyophilisate per mL. The extracts were prepared by dissolving them in 5% [*v/v*] DMSO (CHEMPUR, Piekary Śląskie, Poland). The negative control sample was a 5% [*v/v*] DMSO solution.

The plates were then incubated at the optimum temperature for the growth of the tested microorganisms (37 °C) for 18–24 h. After this time, the zone of inhibition of the growth of the tested *Staphylococcus* bacteria was measured. The result was given in mm ± SD (standard deviation).

The minimum inhibitory concentration (MIC) was determined in a similar manner, as described in Milala et al. [41]. The MIC value in mg/mL was taken as the lowest concentration of the extract at which the zone of inhibition of the growth of the test microorganism was still observed.

### 3.4. Growth of Staphylococcus Bacteria in an Environment with Added Rosa spp. Extracts

In order to assess the effect of rose extracts on the growth of *Staphylococcus* bacteria in a sterile Nutrient Broth medium (Merck, Darmstadt, Germany), *Rosa* spp. extracts were dissolved so that their final concentration in the culture was ¼ MIC for each of these microorganisms (Table 4). The medium prepared in this way was inoculated with a 10% [*v/v*] inoculum of *Staphylococcus* bacteria (density of 0.5–3 according to the McFarland scale). The samples were incubated at 37 °C.

After 0, 4, 8, 16, 24, 60 and 72 h, *Staphylococcus* bacteria were inoculated using the Koch plate method. Nutrient Agar (Merck, Germany) was used to determine the survival of *Staphylococcus* bacteria. The result is given in log*N* ± SD, where *N*—cfu/mL, SD—standard deviation.

Then, the change in the growth/die off rate (µ) of the tested *Staphylococcus* bacteria was determined both in the control culture and in the culture with the addition of extracts.

(1) $$\mu =\frac{1}{N}\frac{dN}{dt}=\frac{N_{t}-N_{0}}{N_{0}(t-t_{0})}$$

where

*µ*—growth or die off rate [h^−1^];

*N_t_*—amount of biomass after the time at which the growth or die-off rate was tested (24 h in the case of determining the growth or die-off rate after 24 h incubation; 72 h in the case of determining the growth or die-off rate in a culture from 48 to 72 h of incubation);

*N*_0_—initial amount of biomass (0 h in the case of determining the growth or die-off rate after 24 h incubation; 24 h in the case of determining the growth or die-off rate in a culture from 48 to 72 h of incubation);

*t*—time after which the growth or die-off rate was tested (24 h in the case of determining the growth/die off rate after 24 h incubation; 72 h in the case of determining the growth or die off rate in a culture from 48 to 72 h of incubation);

*t*_0_—time after which the growth rate was tested or die-off (0 h in the case of determining the rate of growth or die-off after 24 h incubation; 24 h in the case of determining the rate of growth or die-off in culture from 48 to 72 h of incubation).

### 3.5. Scanning Electron Microscopy

Changes in the morphology of *Staphylococcus* bacteria cells under the influence of Rosa spp. extracts were confirmed using scanning electron microscopy.

For this purpose, an electron microscope (JEOL JCM-6000, Tokyo, Japan) was used. The preparations were sputtered with gold (40 s, 8 Pa, 30 mA) using a Joel JFC-1200 Fine Coater (Peabody, MA, USA). Observations were carried out using a voltage of 10 kV and magnifications of 6000 times and 13,000 times.

### 3.6. Statistical Analysis

The presented results are the average value of at least three independent repetitions. The results were also subjected to statistical analysis using the ANOVA test of significance of differences with Tukey’s post hoc test (*p* ≤ 0.05). Calculations were performed in the Statistica 12 program (StatStoft, Cracow, Poland).

For the results of the antagonistic activity of the extracts, the coefficient of determination R^2^ was also determined. The coefficient of determination R^2^ was used for an auxiliary purpose in assessing the proportionality of the antagonistic activity to the concentration of the tested extracts. The R^2^ value was calculated in Excel (Microsoft 365).

## 4. Conclusions

Rose extracts from *Rosa canina*, *Rosa rugosa* and *Rosa pomifera* ‘Karpatia’ may constitute an alternative way to combat coagulase-negative staphylococci isolated from food. This is particularly important in relation to strains that are classified as multidrug-resistant microorganisms (resistant to at least three different classes of antibiotics). The phenomenon of multidrug resistance is increasingly observed among bacteria of the *Staphylococcus* genus that have been isolated from food.

The antagonistic activity of plant extracts, including rose extracts, is often correlated with the concentration used. This work also shows that the higher the concentration of the extract, the larger the growth inhibition zone—this is a positive correlation. It should be noted, however, that it is not linear.

Rose extracts also effectively influence the growth dynamics of *Staphylococcus* bacteria. In most of the cases studied, they act as a factor that, when added to the culture medium, reduces the degree of bacterial cell multiplication in the first 24 h of incubation or significantly extends the adaptation phase. After 72 h of incubation, it is possible to completely inhibit the growth of *Staphylococcus* bacteria by adding extracts from fruit roses to the medium. However, this effect is largely strain-dependent.

SEM analysis confirms that extracts from fruit roses significantly alter the morphology of *Staphylococcus* bacteria. In most cases, the collapse of their centers and changes in the integrity of cell walls are observed, leading to the leakage of intracellular components and cell death. Therefore, the mechanism of the antibacterial action of rose extracts can be attributed to the destruction of the external structures of bacterial cells.

## Figures and Tables

**Table 1:** Dependence of the size of the zone of inhibition of the growth of *Staphylococcus* bacteria on the concentration of *Rosa* spp. extract.

	Zone of Inhibition [mm]						
	*Rosa canina*—Pseudo-Fruit						
Extract Concentration [mg/mL]	ATCC 25923	DSMZ 3270	A5	M5	M6	KR6	KR2A
500	20.50 ± 0.58 ^A^	15.50 ± 0.50 ^A^	24.00 ± 0.82 ^A^	20.33 ± 0.58 ^A^	18.30 ± 0.50 ^A^	18.50 ± 0.50 ^A^	18.00 ± 0.00 ^A^
400	20.00 ± 0.00 ^AB^	14.67 ± 0.58 ^A^	21.00 ± 1.00 ^A^	19.67 ± 0.58 ^A^	17.67 ± 0.58 ^A^	18.00 ± 0.00 ^AB^	16.67 ± 0.58 ^B^
300	19.33 ± 0.58 ^BC^	13.00 ± 0.00 ^B^	20.00 ± 1.73 ^AB^	18.00 ± 0.00 ^B^	17.00 ± 0.00 ^A^	16.67 ± 0.58 ^BC^	15.33 ± 0.58 ^BC^
200	18.33 ± 0.58 ^C^	12.00 ± 0.00 ^B^	20.00 ± 1.73 ^AB^	16.00 ± 0.00 ^C^	16.00 ± 0.00 ^B^	15.33 ± 0.58 ^CD^	15.00 ± 0.00 ^C^
100	16.33 ± 0.58 ^D^	no	19.67 ± 2.31 ^AB^	14.33 ± 0.58 ^D^	15.00 ± 0.00 ^C^	15.00 ± 1.00 ^D^	12.67 ± 0.58 ^C^
50	15.00 ± 0.00 ^E^	no	18.33 ± 1.15 ^ABC^	11.33 ±0.58 ^E^	12.00 ± 0.00 ^D^	14.00 ± 0.00 ^D^	12.33 ± 0.58 ^D^
25	13.67 ± 0.58 ^F^	no	16.37 ± 0.58 ^BC^	no	no	no	no
12.5	12.00 ± 0.00 ^G^	no	16.33 ± 0.58 ^BC^	no	no	no	no
6.25	no	no	16.67 ± 0.58 ^BC^	no	no	no	no
3.13	no	no	14.33 ± 1.53 ^C^	no	no	no	no
1.56	no	no	no	no	no	no	no
	***Rosa canina* flesh (rose flesh)**						
500	14.33 ± 1.89 ^A^	12.33 ± 0.58 ^A^	15.33 ± 1.50 ^A^	17.50 ± 0.50 ^A^	11.80 ± 0.50 ^A^	17.33 ± 0.58 ^A^	14.50 ± 1.29 ^A^
400	13.67 ± 0.58 ^A^	11.00 ± 0.00 ^B^	14.00 ± 0.00 ^A^	14.67 ± 0.58 ^B^	11.00 ± 0.00 ^A^	14.33 ± 1.15 ^B^	12.67 ± 0.58 ^AB^
300	12.33 ± 0.58 ^A^	no	13.00 ± 0.00 ^B^	14.33 ± 0.58 ^B^	no	14.33 ± 0.58 ^B^	12.00 ± 0.00 ^B^
200	no	no	no	11.00 ± 0.00 ^C^	no	12.67 ± 0.58 ^C^	no
100	no	no	no	no	no	no	no
50	no	no	no	no	no	no	no
25	no	no	no	no	no	no	no
12.5	no	no	no	no	no	no	no
6.25	no	no	no	no	no	no	no
3.13	no	no	no	no	no	no	no
1.56	no	no	no	no	no	no	no
	***Rosa rugosa*—pseudo-fruit**						
500	23.80 ± 0.96 ^A^	22.50 ± 2.38 ^A^	28.50 ± 1.29 ^A^	24.00 ± 0.82 ^A^	20.00 ± 0.82 ^A^	20.33 ± 0.96 ^A^	21.33 ± 0.96 ^A^
400	22.67 ± 0.58 ^A^	20.67 ± 0.58 ^A^	26.33 ± 0.58 ^A^	21.67 ± 0.58 ^B^	19.67 ± 0.58 ^A^	20.33 ± 0.58 ^AB^	21.00 ± 0.00 ^A^
300	21.00 ± 0.00 ^B^	19.67 ± 0.58 ^A^	24.67 ± 0.58 ^B^	20.67 ± 0.58 ^BC^	18.67 ± 0.58 ^A^	20.00 ± 0.00 ^AB^	20.00 ± 0.00 ^A^
200	20.00 ± 0.00 ^BC^	17.67 ± 0.58 ^B^	23.67 ± 0.58 ^BC^	20.33 ± 0.58 ^CD^	15.67 ± 0.58 ^B^	19.33 ± 0.58 ^B^	19.67 ± 0.58 ^A^
100	19.67 ±0.58 ^BC^	16.33 ± 0.58 ^BC^	22.67 ± 0.58 ^C^	19.33 ± 0.58 ^D^	14.67 ± 0.58 ^B^	18.00 ± 0.00 ^C^	19.33 ± 0.58 ^A^
50	19.00 ± 0.00 ^C^	15.67 ± 0.58 ^C^	21.00 ± 0.00 ^D^	16.67 ± 0.58 ^E^	no	17.00 ± 0.00 ^C^	16.33 ± 0.58 ^B^
25	16.67 ± 0.58 ^D^	13.67 ± 0.58 ^D^	20.00 ± 0.00 ^DE^	15.00 ± 0.00 ^F^	no	15.00 ± 0.00 ^D^	16.00 ± 0.00 ^B^
12.5	15.33 ± 0.58 ^D^	no	20.00 ± 0.00 ^DE^	13.00 ± 0.00 ^G^	no	12.67 ± 0.58 ^E^	15.33 ± 0.58 ^B^
6.25	13.33 ± 0.58 ^E^	no	19.00 ± 0.00 ^E^	no	no	no	13.00 ± 0.00 ^C^
3.13	no	no	no	no	no	no	no
1.56	no	no	no	no	no	no	no
	***Rosa rugosa*—flesh (rose flesh)**						
500	13.00 ± 0.00 ^A^	14.00 ± 1.15 ^A^	13.50 ± 0.50 ^A^	23.00 ± 0.00 ^A^	11.50 ± 0.50 ^A^	18.33 ± 1.53 ^A^	14.50 ± 0.50 ^A^
400	no	12.00 ± 0.00 ^A^	12.67 ± 0.58 ^A^	21.00 ± 0.00 ^B^	no	17.67 ± 1.53 ^A^	12.00 ± 0.00 ^B^
300	no	no	no	19.00 ± 0.00 ^C^	no	15.00 ± 1.00 ^B^	no
200	no	no	no	18.00 ± 0.00 ^D^	no	14.00 ± 0.00 ^BC^	no
100	no	no	no	16.00 ± 0.00 ^E^	no	13.00 ± 0.00 ^BC^	no
50	no	no	no	15.00 ± 0.00 ^F^	no	12.00 ± 0.00 ^C^	no
25	no	no	no	14.00 ± 0.00 ^G^	no	no	no
12.5	no	no	no	12.00 ± 0.00 ^H^	no	no	no
6.25	no	no	no	no	no	no	no
3.13	no	no	no	no	no	no	no
1.56	no	no	no	no	no	no	no
	***Rosa pomifera*** **‘Karpatia’—pseudo-fruit**						
500	20.00 ± 1.83 ^A^	18.80 ± 0.96 ^A^	22.80 ± 0.96 ^A^	23.00 ± 0.00 ^A^	18.50 ± 1.29 ^A^	20.70 ± 0.58 ^A^	19.00 ± 1.83 ^A^
400	19.67 ± 0.58 ^A^	16.33 ± 0.58 ^B^	20.33 ± 0.58 ^B^	22.00 ± 0.00 ^B^	17.67 ± 0.58 ^A^	18.67 ± 0.58 ^B^	17.33 ± 0.58 ^B^
300	18.00 ± 0.00 ^B^	14.00 ± 0.00 ^C^	19.67 ± 0.58 ^BC^	20.67 ± 0.58 ^C^	15.33 ± 0.58 ^AB^	17.67 ± 0.58 ^B^	16.00 ± 0.00 ^C^
200	17.00 ± 0.00 ^B^	no	19.00 ± 0.00 ^BCD^	19.00 ± 0.00 ^D^	14.33 ± 0.58 ^BC^	15.67 ± 0.58 ^C^	14.67 ± 0.58 ^D^
100	15.67 ± 0.58 ^C^	no	18.67 ± 0.58 ^CD^	17.67 ± 0.58 ^E^	13.33 ± 0.58 ^C^	15.00 ± 0.00 ^CD^	13.67 ± 0.58 ^D^
50	14.67 ± 0.58 ^C^	no	18.00 ± 0.00 ^CD^	14.67 ± 0.58 ^F^	no	14.33 ± 0.58 ^CD^	no
25	13.00 ± 0.00 ^D^	no	17.67 ± 0.58 ^DE^	13.00 ± 0.00 ^G^	no	14.00 ± 0.00 ^D^	no
12.5	no	no	17.00 ± 0.00 ^DE^	12.00 ± 0.00 ^H^	no	no	no
6.25	no	no	14.67 ± 0.58 ^E^	no	no	no	no
3.13	no	no	13.67 ± 0.58 ^F^	no	no	no	no
1.56	no	no	no	no	no	no	no
	***Rosa pomifera* ‘Karpatia’—flesh (rose flesh)**						
500	13.00 ± 0.00 ^A^	13.00 ± 0.00 ^A^	18.50 ± 1.29 ^A^	19.80 ± 0.96 ^A^	13.00 ± 0.82 ^A^	16.50 ± 173 ^A^	13.00 ± 0.00 ^A^
400	no	12.00 ± 0.00 ^B^	no	19.33 ± 0.58 ^A^	no	14.67 ± 0.58 ^A^	no
300	no	no	no	18.00 ± 0.58 ^B^	no	13.67 ± 0.58 ^A^	no
200	no	no	no	17.67 ± 0.58 ^B^	no	no	no
100	no	no	no	16.00 ± 0.00 ^C^	no	no	no
50	no	no	no	15.00 ± 0.58 ^CD^	no	no	no
25	no	no	no	14.67 ± 0.58 ^D^	no	no	no
12.5	no	no	no	13.00 ± 0.00 ^E^	no	no	no
6.25	no	no	no	no	no	no	no
3.13	no	no	no	no	no	no	no
1.56	no	no	no	no	no	no	no

no—growth inhibition not observed. Table shows mean values ± standard deviation; A–H—mean values denoted by different letters differ statistically significantly (ANOVA, post hoc Tukey test *p* ≤ 0.05). Tested bacteria: ATCC 25923—*Staphylococcus aureus* ATCC 25923, DSMZ 3270—*Staphylococcus epidermidis* DSMZ 3270, A5—*Staphylococcus epidermidis* A5, M5—*Staphylococcus xylosus* M5, M6—*Staphylococcus haemolyticus* M6, KR6—*Staphylococcus capitis* KR6, KR2A—*Staphylococcus warneri* KR2A.

**Table 2:** Coefficient of determination in the assessment of the antagonistic activity of the tested extracts.

*Staphylococcus* Strain	R^2^ Coefficient for Linear Trend Line					
	RCC	RCM	RRC	RRM	RKC	RKM
ATCC 25923	0.4337	X	0.3547	X	0.4358	X
DSMZ 3270	0.7339	X	0.4332	X	X	X
A5	0.2521	X	0.2949	X	0.2305	X
M5	0.6079	0.7941	0.4050	0.4730	0.5224	0.3619
M6	0.4699	X	0.5981	X	0.5870	X
KR6	0.4167	0.6740	0.3585	0.5351	0.3743	X
KR2A	0.4757	X	0.2756	X	0.5480	X
	**R^2^ for Logarithmic Trend Line**					
	**RCC**	**RCM**	**RRC**	**RRM**	**RKC**	**RKM**
ATCC 25923	0.7786	X	0.7381	X	0.7419	X
DSMZ 3270	0.8812	X	0.7330	X	X	X
A5	0.6204	X	0.6126	X	0.6213	X
M5	0.8633	0.9232	0.7795	0.7859	0.8425	0.6913
M6	0.7697	X	0.8096	X	0.8033	X
KR6	0.6865	0.8356	0.7372	0.7792	0.6515	X
KR2A	0.7489	X	0.6916	X	0.7758	X

X—calculation impossible. Tested bacteria: ATCC 25923—*Staphylococcus aureus* ATCC 25923, DSMZ 3270—*Staphylococcus epidermidis* DSMZ 3270, A5—*Staphylococcus epidermidis* A5, M5—*Staphylococcus xylosus* M5, M6—*Staphylococcus haemolyticus* M6, KR6—*Staphylococcus capitis* KR6, KR2A—*Staphylococcus warneri* KR2A. Tested cultures: RCC—cultivation with the addition of extract from the whole pseudo-fruit of *Rosa canina*; RCM—cultivation with the addition of *Rosa canina* flesh extract; RRC—cultivation with the addition of extract from the whole pseudo-fruit of *Rosa rugosa*; RRM—cultivation with the addition of *Rosa rugosa* flesh extract; RKC—cultivation with the addition of extract from the whole pseudo-fruit of *Rosa pomifera* ‘Karpatia’; RKM—cultivation with the addition of *Rosa pomifera* ‘Karpatia’ flesh extract.

**Table 3:** Change in the growth/death rate of the tested *Staphylococcus* strains under the influence of *Rosa* spp. extracts.

*Staphylococcus* Strain	∆μ [h^−1^]			
	Culture Without Extract Addition			
	0–24 h		24–72 h	
ATCC 25923	0.006		0.001 *	
DSMZ 3270	0.009		0.002 *	
A5	0.007		0.001 *	
M5	0.006		0.001 *	
M6	0.010		0.001 *	
KR6	0.015		0.003 *	
KR2A	0.012		0.001 *	
***Staphylococcus* Strain**	**∆µ [h^−1^]**			
	**Culture with Addition of *Rosa canina* Extract**			
	**Flesh (Rose Flesh)**		**Whole Pseudo-Fruit**	
	**0–24 h**	**24–72 h**	**0–24 h**	**24–72 h**
ATCC 25923	0.008 *	0.009 *	0.004	0.001
DSMZ 3270	0.014 *	0.004 *	0.009 *	0.006 *
A5	0.011 *	0.021 *	0.005	0.001
M5	0.001 *	0.010 *	0.006	0.000
M6	0.010 *	0.021 *	0.008	0.021 *
KR6	0.009 *	0.007 *	0.009	0.000
KR2A	0.008 *	0.008 *	0.012 *	0.007 *
***Staphylococcus* Strain**	**∆µ [h^−1^]**			
	**Culture with Addition of *Rosa rugosa* Extract**			
	**Flesh (Rose Flesh)**		**Whole Pseudo-Fruit**	
	**0–24 h**	**24–72 h**	**0–24 h**	**24–72 h**
ATCC 25923	0.011 *	0.013 *	0.004	0.002 *
DSMZ 3270	0.011 *	0.008 *	0.005	0.000
A5	0.008 *	0.007 *	0.006	0.001
M5	0.009	0.001 *	0.009	0.001 *
M6	0.017 *	0.021 *	0.009 *	0.005 *
KR6	0.008 *	0.004 *	0.012 *	0.010 *
KR2A	0.007 *	0.009 *	0.005	0.001 *
***Staphylococcus* Strain**	**∆µ [h^−1^]**			
	**Culture with Addition of *Rosa pomifera* ‘Karpatia’ Extract**			
	**Flesh (Rose Flesh)**		**Whole Pseudo-Fruit**	
	**0–24 h**	**24–72 h**	**0–24 h**	**24–72 h**
ATCC 25923	0.001	0.002 *	0.008	0.004 *
DSMZ 3270	0.014 *	0.007 *	0.021 *	0.002 *
A5	0.008 *	0.009 *	0.008	0.001 *
M5	0.007	0.001 *	0.008	0.001 *
M6	0.019 *	0.005 *	0.003 *	0.005 *
KR6	0.015 *	0.004 *	0.003 *	0.004 *
KR2A	0.016 *	0.002 *	0.14 *	0.05 *

*—death rate. Tested bacteria: ATCC 25923—*Staphylococcus aureus* ATCC 25923, DSMZ 3270—*Staphylococcus epidermidis* DSMZ 3270, A5—*Staphylococcus epidermidis* A5, M5—*Staphylococcus xylosus* M5, M6—*Staphylococcus haemolyticus* M6, KR6—*Staphylococcus capitis* KR6, KR2A—*Staphylococcus warneri* KR2A.

**Table 4:** Concentration of *Rosa* spp. extracts used in the study of growth dynamics.

*Staphylococcus* Strain	Extract Concentration [mg/mL]					
	*Rosa canina*		*Rosa rugosa*		*Rosa pomifera* ‘Karpatia’	
	RCC	RCM	RRC	RRM	RKC	RKM
ATCC 25923	3.125	75	0.781	125	6.25	125
DSMZ 3270	50	125	6.25	100	75	100
A5	0.781	75	1.563	100	0.781	25
M5	12.5	75	1.563	3.125	3.125	12.5
M6	12.5	125	25	125	25	100
KR6	6.25	50	3.125	12.5	6.25	75
KR2A	6.25	75	1.563	100	25	125

Tested bacteria of the *Staphylococcus genus*: *Staphylococcus aureus* ATCC 25923, *Staphylococcus epidermidis* DSMZ 3270, *Staphylococcus epidermidis* A5, *Staphylococcus xylosus* M5, *Staphylococcus haemolyticus* M6, *Staphylococcus capitis* KR6, *Staphylococcus warneri* KR2A. RCC—extract from the whole pseudo-fruit of *Rosa canina*; RCM—*Rosa canina* flesh extract; RRC—extract from the whole pseudo-fruit of *Rosa rugosa*; RRM—*Rosa rugosa* flesh extract; RKC—extract from the whole pseudo-fruit of *Rosa pomifera* ‘Karpatia’; RKM—*Rosa pomifera* ‘Karpatia’ flesh extract.

## Data Availability

The data presented in this study are available on request from the corresponding author.

## Abbreviations

The following abbreviations are used in this manuscript:

MIC	Minimal inhibitory concentration
SEM	Scanning electron microscopy
RCC	Extract from the whole pseudo-fruit of *Rosa canina*
RCM	*Rosa canina* flesh extract
RRC	Extract from the whole pseudo-fruit of *Rosa rugosa*
RRM	*Rosa rugosa* flesh extract
RKC	Extract from the whole pseudo-fruit of *Rosa pomifera* ‘Karpatia’
RKM	*Rosa pomifera* ‘Karpatia’ flesh extract
